# Comparative Effectiveness of Combined IgM-Enriched Immunoglobulin and Extracorporeal Blood Purification Plus Standard Care Versus Standard Care for Sepsis and Septic Shock after Cardiac Surgery

**DOI:** 10.31083/j.rcm2309314

**Published:** 2022-09-14

**Authors:** Gianluca Paternoster, Silvia De Rosa, Pietro Bertini, Pasquale Innelli, Rosaria Vignale, Vincenzo Francesco Tripodi, Giuseppe Buscaglia, Mariacristina Vadalà, Michele Rossi, Antonio Arena, Andrea Demartini, Giovanni Tripepi, Domenico Abelardo, Giuseppe Pittella, Aldo Di Fazio, Sabino Scolletta, Fabio Guarracino, Blanca Martinez Lopez de Arroyabe

**Affiliations:** ^1^Cardiovascular Anesthesia and ICU, San Carlo Hospital, 85100 Potenza, Italy; ^2^Department of Anesthesia and Intensive Care Unit, San Bortolo Hospital, 36100 Vicenza, Italy; ^3^Department of Aaesthesia and Critical Care Medicine, Azienda Ospedaliero-Universitaria Pisana, 56126 Pisa, Italy; ^4^Intensive Cardiac Care Unit, San Carlo Hospital, 85100 Potenza, Italy; ^5^CardioThoracoVascular Department, Heart Center, Grande Ospedale Metropolitano “Bianchi-Melacrino-Morelli”, 89124 Reggio Calabria, Italy; ^6^Caridiovascular Anesthesia and ICU, Ospedale san Martino, 16132 Genova, Italy; ^7^Institute of Clinical Physiology (IFC-CNR), Clinical Epidemiology, And Physiopathology of Renal Diseases and Hypertension of Reggio Calabria, 89124 Reggio Calabria, Italy; ^8^Regional Complex Intercompany Institute of Legal Medicine, San Carlo Hospital, 85100 Potenza, Italy; ^9^Department of Emergency and Organ Transplant, University of Siena, 53100 Siena, Italy; ^10^Cardiothoracic and Vascular Anesthesia and Intensive Care University Hospital, 37126 Verona, Italy

**Keywords:** blood purification therapy, cardiac surgery, IgM-enriched immunoglobulin, sepsis, pentaglobin, hyper-inflammation, immunosuppression

## Abstract

**Background:**

The combination of surgery, bacterial spread-out, and artificial cardiopulmonary bypass surfaces results in a release of key inflammatory mediators leading to an overshooting systemic hyper-inflammatory condition frequently associated with compromised hemodynamics and organ dysfunction. A promising approach could be extracorporeal blood purification therapies in combination with IgM enriched immunoglobulin. This approach might perform a balanced control of both hyper and hypo-inflammatory phases as an immune-modulating intervention.

**Methods:**

We performed a retrospective observational study of patients with proven infection after cardiac surgery between January 2020 and December 2021. Patients were divided into two groups: (1) the first group (Control Group) followed a standard care approach as recommended by the Surviving Sepsis Campaign Guidelines; The second group (Active Group) underwent extracorporeal blood purification therapy (EBPT) in combination with intravenous administration of IgM enriched immunoglobulin 5 mL/kg die for at least three consecutive days, in conjunction with the standard approach (SSC Guidelines). In addition, ventriculo-arterial (V/A) coupling, Interleukin 6 (IL-6), Endotoxin Activity Assay (EAA), Procalcitonin, White Blood Cells (WBC) counts, Sequential Organ Failure Assessment (SOFA) Score and Inotropic Score were assessed in both two groups at different time points.

**Results:**

Fifty-four patients were recruited; 25 were in the Control Group, while 29 participants were in the Active Group. SOFA score significantly improved from baseline [12 (9–16)] until at $T_{3}$ [8 (3–13)] in the active group; it was associated with a median EAA reduction from 1.03 (0.39–1.20) at $T_{0}$ to 0.41 (0.2–0.9) at $T_{3}$ in the active group compared with control group 0.70 (0.50–1.00) at $T_{0}$ to 0.70 (0.50–1.00) at $T_{3}$ (*p *< 0.001). V/A coupling tended to be lower in patients of the active arm ranging from 1.9 (1.2–2.7) at $T_{0}$ to 0.8 (0.8–2.2) at $T_{3}$ than in those of the control arm ranging from 2.1 (1.4–2.2) at T0 to 1.75 (1.45–2.1) at $T_{3}$ (*p* = 0.099). The hemodynamic improvement over time was associated with evident but no significant decrease in inotropic score in the active group compared with the control group. Changes in EAA value from $T_{0}$ to $T_{4}$ were directly and significantly related (r = 0.39, *p* = 0.006) to those of V/A coupling.

**Conclusions:**

EBPT, in combination with IgM enriched immunoglobulin, was associated with a mitigated postoperative response of key cytokines with a significant decrease in IL-6, Procalcitonin, and EAA and was associated with improvement of clinical and metabolic parameters.

## 1. Introduction

Sepsis is a potentially life-threatening condition caused by an infection and an inadequate dysregulation of host immune response [1]. Sepsis is one of the leading causes of mortality despite the extensive efforts and many different types of treatments [2].

In cardiac surgery, the prevalence of sepsis is between 0.39% and 2.5% [3], with a mortality ranging from 65% up to 79% [4, 5]. However, myocardial dysfunction, characterized by biventricular dilatation and reduced ejection fraction, is present in most septic patients, and it seems to be not due to myocardial hypoperfusion but to circulating depressant factors; including the cytokines tumor necrosis factor-alpha and IL-1β [6, 7, 8, 9, 10].

Notably, during the perioperative period in cardiac surgery, many factors such as surgical trauma, shear stress, blood contact with cardiopulmonary bypass (CBP), internal drainage system, blood transfusion, and reperfusion after ischemia could influence and impact patients’ outcomes. In addition, cardiac surgery with cardiopulmonary bypass is associated with gut barrier dysfunction and endotoxin lipopolysaccharide (LPS) release. These factors together can provoke a dynamic systemic immune response. Therefore, several new immunomodulatory approaches have been investigated during the last years, among them the immunostimulation and the extracorporeal blood purification techniques (EBPTs) [11, 12, 13, 14, 15, 16].

Several studies and case series supported the use of Polyvalent intravenous Immunoglobulins and blood purification based on pleiotropic effects on the inflammatory and immune mechanisms and the beneficial effects on hemodynamic and survival [17, 18, 19, 20, 21, 22, 23, 24].

Although the evidence for beneficial effects of IgM-enriched Immunoglobulins in patients with severe sepsis and septic shock has not always been supportive, systematic reviews have generally concluded that IgM-enriched immunoglobulin preparations are associated with a reduction in mortality [25, 26, 27, 28, 29, 30].

Extracorporeal blood purification techniques have a history of 15 years in treating critically ill patients [31, 32]. Removing or decreasing serum concentration of inflammatory mediators, fragments of gut-derived Gram-negative (lipopolysaccharides or endotoxin) and tissue degradation products from the systemic circulation can provide beneficial effects (preventing multi-organ dysfunction and immune-paralysis) [33, 34, 35, 36, 37, 38, 39, 40, 41, 42, 43, 44].

However, we still need further studies to establish the appropriate technique, patient selection, timing, duration of the treatment, and the effect on solid clinical endpoints (mortality, organ dysfunction).

The purpose of the present study was to assess the effectiveness of combined IgM-enriched Immunoglobulin and extracorporeal blood purification plus standard care versus standard care alone for sepsis and septic shock after cardiac surgery.

We hypothesize that blood purification therapies combined with IgM-enriched Immunoglobulins used as adjunctive therapy may reduce significant cytokine concentrations and improve hemodynamic stability.

As a marker of the cardiovascular system balance, we used the ratio between arterial elastance (Ea) and left ventricular end-systolic elastance (Ees), called ventriculo-arterial coupling. It was used and investigated in the septic shock population [45, 46].

## 2. Materials and Methods

### 2.1 Study Design and Setting

This investigation was a retrospective, observational study. Data were collected retrospectively on patients admitted into the Cardiac Surgery Intensive Care Units (CSICU) from January 2020 to December 2021. The San Carlo Hospital Ethics Committee, Potenza, Italy, has approved the protocol. Therefore, the need for informed consent was waived. The study was designed following the Declaration of Helsinki.

### 2.2 Study Population

Patients with a diagnosis of sepsis and septic shock were identified according to Surviving Sepsis Campaign 2016 criteria [47, 38]. Subjects who satisfied the following requirements within 24 hours and had a known or suspected infection based on clinical data at the time of screening were included in the study: three or more indicators of systemic inflammation and at least two organs or organ systems that are dysfunctional as a result of sepsis. Patients who were expected to die within 28 days due to an untreatable medical condition, such as a poorly controlled neoplasm or other moribund state end-stage diseases in which death was thought to be imminent, were excluded from the analysis, as pregnant or nursing women and patients under the age of 18.

Patients were categorized into two groups: (1) Active group: septic patients underwent extracorporeal blood purification in combination with intravenous administration of IgM enriched Immunoglobulin (Pentaglobin®) 5 mL/kg die for at least three consecutive days and in conjunction with standard care approach under the Surviving Sepsis Campaign Guidelines; (2) Control group: a standard care approach following the Surviving Sepsis Campaign Guidelines.

### 2.3 Variables and Measurements

Data were prospectively collected during the patient’s admission and entered into a database for research purposes. All patients’ demographic data were obtained retroactively. Cardiothoracic surgery techniques included valve surgery, coronary artery bypass graft (CABG), or combined surgery. The latter category comprises several complex procedures such as surgery for congenital heart diseases, aortic aneurysms, and aortic dissections. V/A coupling and Inotropic score were used to evaluate the cardiovascular system function. A specially developed program (iElastance - an Apple iOS App) for measuring non-invasive single beat end-systolic Ees by the Chen method [48] and Ea as systolic blood pressure multiplied by 0.9/SV was used to calculate the V/A (Ea/Ees ratio) [49, 50]. The Doppler velocity-time integral (VTI) approach was applied to measure stroke volume (SV) as left ventricular outflow tract (LVOT) area × LVOT VTI. In the parasternal long-axis perspective, the LVOT area was calculated as LVOT2 ×/4 = LVOT2 × 0.785. The zoom feature was used to obtain the LVOT image, and the leaflets’ immediate surroundings were measured “inner-edge to inner-edge” in mid-systole. The apical 5-chamber was used to record the LVOT velocity, and the sample volume was placed roughly where the 2D LVOT measurements were made—about 5 mm from the aortic valve. When the sample volume was positioned correctly, the signal’s spectral broadening or the aortic valve’s closing click could be observed. As a 2D technique, the biplane method of disks (a modified version of Simpson’s rule) was employed to measure left ventricular ejection fraction (LVEF) [46]. Pre-ejection time and total ejection time were measured from the beginning of the electrocardiographic QRS complex until the beginning of the aortic flow and to the end of the aortic flow, respectively. Mean arterial pressure was also used to evaluate the hemodynamic state (MAP). The inotrope score was calculated using the following formula: dopamine (dose × 1)  +  dobutamine (dose × 1)  +  amrinone (dose × 1)  +  milrinone (dose × 15)  +  epinephrine (dose × 100) +  norepinephrine (dose × 100)  + enoximone (dose × 1)  +  isoprenaline (dose × 100), with dose in μg/kg/min. Organ dysfunction was assessed by the Sequential Organ Failure Assessment (SOFA) score [51]. Data obtained on admission in ICU (T0) and at three different timepoints (24, 48 and 72 hours after ICU admission) were collected and compared between the two groups, including Interleukin 6 (IL-6), Endotoxin Activity Assay (EAA), Procalcitonin, White Blood Cells (WBC) count, SOFA Score and Inotropic Score were calculated at different time points: at baseline, before treatment (T0), 24 hrs after treatment (T1); 48 hrs after treatment (T2); 72 hrs after treatment (T3). A secondary endpoint was 28-day survival.

#### 2.3.1 Endotoxin Activity Analysis

By using the EAA (Spectral Diagnostics Inc., Toronto, ON, Canada), a quick 30-minutes *in vitro* test that assesses neutrophil response to endotoxin by chemiluminescent reaction, blood endotoxin activity was determined. Sepsis risk and poorer clinical outcomes, both at ICU discharge and hospital discharge, were both related to EAA 0.4 [11, 52].

#### 2.3.2 Endpoint

Assessment of multiorgan dysfunction (SOFA) and the dynamics of biochemical and biohumoral variables (EAA, PCT, IL-6, and WBC) over time served as the study’s primary endpoints. Hospital mortality in both groups and the advancement of hemodynamic dysfunction and ventricular-arterial coupling, both viewed as performance indicators of the cardiovascular system, were secondary endpoints.

#### 2.3.3 Statistical Analysis

Continuous variables were reported as median and interquartile range [IQR]. 25th–75th quartiles range (IQR). Categorical variables were expressed as absolute number (N) and percentage (%). As appropriate, between groups, comparisons were performed by independent *T*-Test, Mann-Whitney Test, or Chi-Square Test. The relationship between the two variables was assessed by calculating the Pearson product moment correlation coefficient (r) and *p* value. The effect of the allocation arm (active group versus control group) on the evolution over time of repeated measurements of biomarkers and the SOFA score was investigated by the Generalised Linear Model (GLM). In these models, data were adjusted for the corresponding baseline value of each variable. For descriptive purposes, the evolution of key variables over time was reported as mean and 95% CI. The Kaplan-Meier analysis and log-rank test carried out the time-to-death analysis. Data analysis was carried out by SPSS for Windows, version 22, IBM, Chicago, IL, USA.

#### 2.3.4 Ethical Concerns

The study did not involve medical, pharmacological, or behavioral interventions in addition to hospital standards of care. This research has been carried out in agreement with the principles laid out in the original Declaration of Helsinki and its later amendments. All patients included in the article provided informed consent.

## 3. Results

### 3.1 Baseline and Clinical Characteristics

Fifty-four hospitalized patients were included in the study (56% males, mean age 63 ± 13 in the active group and 68 ± 11 in the control group). Patients’ demographics, age, sex, comorbidities, and clinical characteristics are shown in Table 1.

**Table 1.** **Main characteristics of the study population. The table shows comorbidities, the demographic and baseline clinical data of the study**.

Baseline variables		Entire cohort	Active group	Control group	*p* value
		(n = 54)	(n = 29)	(n = 25)	
Age (years)		65 ± 12	63 ± 13	68 ± 11	0.12
Male gender n. (%)		30 (56)	18 (62)	12 (48)	0.30
Renal insufficiency n. (%)		18 (33)	11 (38)	7 (28)	0.44
SOFA score (points)		10 (9–13)	12 (10–13)	9 (8–9)	<0.001
Ejection fraction (%)		45.0 ± 10.3	44.6 ± 11.1	45.5 ± 9.5	0.76
CBP time (min)		96.9 ± 33.7	96.9 ± 43.2	96.9 ± 18.3	0.99
Aortic Cross Clamp Time (min)		78.9 ± 26.5	76.7 ± 33.9	81.3 ± 14.0	0.51
V/A coupling		1.9 ± 0.4	1.9 ± 0.4	2.0 ± 0.3	0.53
Mean arterial pressure (mmHg)		63.3 ± 7.1	62.6 ± 7.6	64.2 ± 6.4	0.41
Inotropic score		13.9 ± 4.8	14.3 ± 5.4	13.4 ± 4.0	0.52
EAA		0.9 ± 0.3	1.0 ± 0.4	0.8 ± 0.2	0.002
PCT (ng/mL)		7.4 (5.0–12.2)	7.0 (5.0–11.5)	7.9 (5.0–13.0)	0.81
IL-6 (pg/mL)		76.1 ± 19.8	78.0 ± 16.7	73.9 ± 22.9	0.46
WBC (×${\mathrm{10}}^{\wedge }$3/uL)		17.6 ± 9	20.0 ± 10.3	14.8 ± 6.5	0.03
AST U/L		32.6 ± 11.7	34.9 ± 12.1	31.3 ± 13.4	0.35
ALT U/L		36.37 ± 12.6	41.4 ± 11.2	32.4 ± 12.1	0.42
Albumin g/L		28.1 ± 5.4	29.2 ± 4.5	27.8 ± 5–6	0.43
Type of surgery					
	Valve	27	15 (62.5%)	12 (37.5%)	
	CABG	15	8 (53.3%)	7 (46.7%)	
	Combined	10	3 (30%)	7 (70%)	
	Ascending Aorta	2	2 (100%)	0 (0%)	

V/A coupling, ventricular arterial copling; CBP time, Cardioplumonary bypass time; EAA, endotoxin activity assay; PCT, procalcitonon; IL-6, interleukin-6; WBC, white blood cells; AST, asparatate aminotransferase; ALT, alanine aminotrasferase.

Colonization was present in 6 (21%) patients in the active group compared to 3 (12%) in the control group (*p* = 0.48). Multiple drug resistance (MDR) Bacteria in the active group were 12 (41%) compared to 7 (28%) in the control group (*p* = 0.34). Considering the whole population, Methicillin-Resistant Staphylococcus Aureus (MRSA) was present in one patient (3.4%) in the active group. Microbiological identification of bacterial micro-organisms in both groups is reported in Table 2. Among 54 patients, 29 were on the standard treatment plus blood purification and IgM enriched Immunoglobulins (active group), and the remaining 25 patients with standard treatment alone (control group). In the active group, one patient (3.5%) was treated with Coupled Plasma Filtration and Adsorption (CPFA) together with Cytosorb® Cartridge and Toraymyxin® Cartridge 4 patients (13.8%) were treated with continuous venovenous hemodiafiltration (CVVHDF), 4 (13.8%) were treated with Cytosorb® Cartridge, 7 (24%) were treated with Ultraflux ® EMIC2 Filter, and 13 (45%) were treated with Toraymyxin® Cartridge.

**Table 2.** **Pathogens isolated in the two groups**.

Pathogen	Entire cohort	Active group	Control group
	(n = 54)	(n = 29)	(n = 25)
Acinetobacter Baumani	12	6	6
Acinetobacter Baumani plus Klebsiella pneumoniae	1	0	1
Enterobacter cloacae	1	0	1
Enterococcus faecium	3	2	1
Klebsiella Pneumoniae	11	8	3
Moraxella	1	0	1
Pseudomonas Aeurginosa	6	2	4
Staphylococcus Aureus plus Acinetobac Baumanii	5	3	2
Staphylococcus Aureus	6	4	2
Staphylococcus Meticillin resistant	2	1	1
Staphylococcus Meticililin Resistant plus Acinetobacter Baumanii	1	0	1
Streptococco Equisimilis	2	1	1
None	1	1	0

The two groups did not significantly differ in age and gender as well as in the prevalence of renal insufficiency, ejection fraction, CBP and Aortic Cross Clamp Times, MAP, and inotropic score (Table 1). At baseline, circulating levels of PCT and IL-6 were similar between the two groups. However, the SOFA score, WBC, and EAA were significantly higher in patients of the active group than in the control group (Table 1). The pathogens isolated in patients of the two groups are given in Table 2.

### 3.2 SOFA Score and Biomarkers over Time

**Fig. 1.**
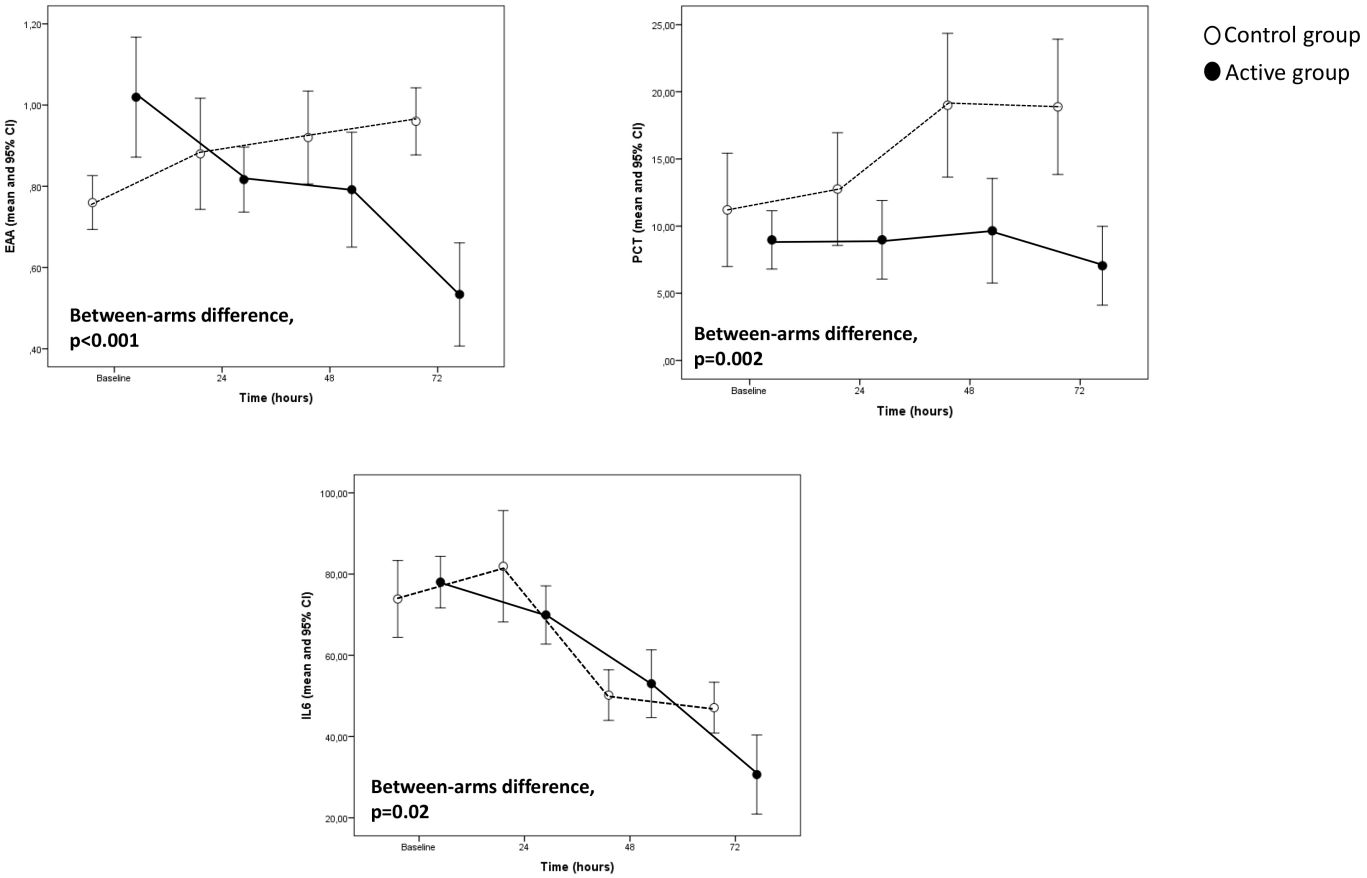
**Major biomarkers trend in the study population**.

**Fig. 2.**
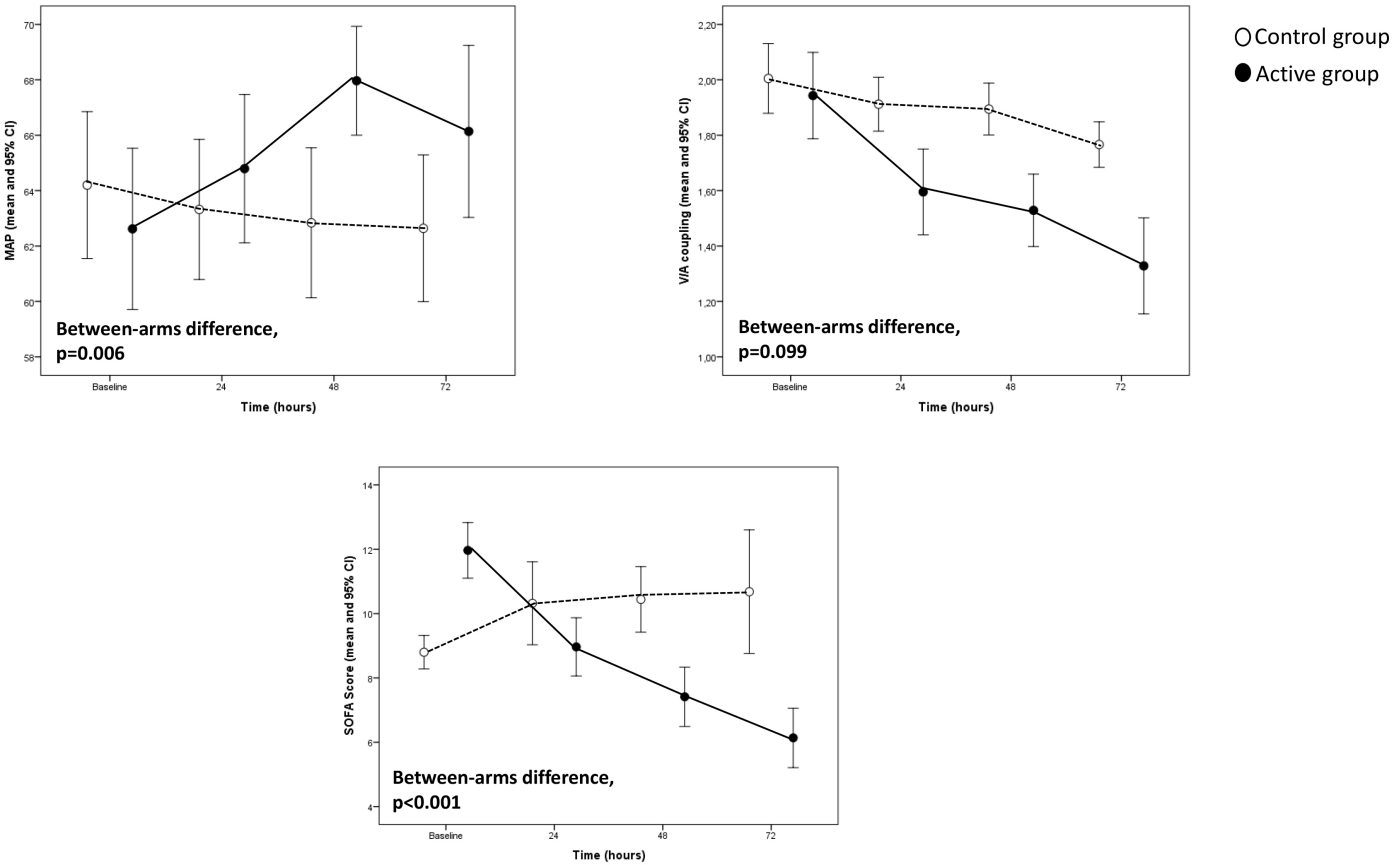
**Hemodynamic recovery variables and sofa score trends in the study population**.

**Fig. 3.**
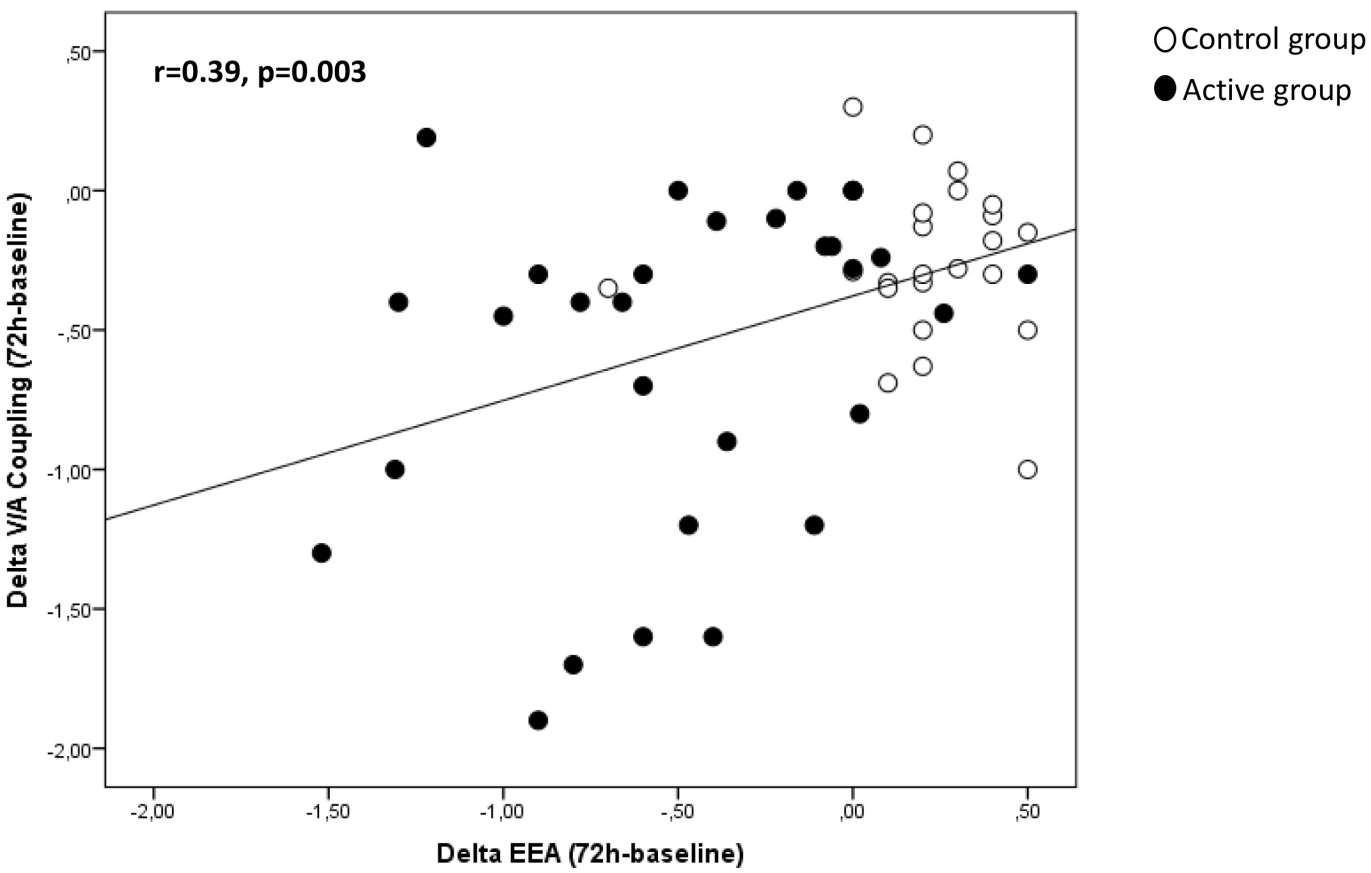
**Correlation between EAA and V/A coupling in the study population**.

In the Generalised Linear Model adjusted for the corresponding baseline value, the evolution over time of EEA (*p *< 0.001), PCT (*p* = 0.002), and IL-6 (*p* = 0.02) (see Fig. 1) as well as SOFA score (*p *< 0.001) (see Fig. 2) maintained significantly lower in patients of the active group than in those of the control group whereas MAP displayed an opposite pattern (see Fig. 2). In the same analysis, V/A coupling tended to be lower (*p* = 0.099) in patients of the active arm than in those of the control am. Of note, the changes of EEA between baseline and 72 h were directly and significantly related (r = 0.39, *p* = 0.006) to those of V/A coupling (Fig. 3).

### 3.3 Survival Analysis

**Fig. 4.**
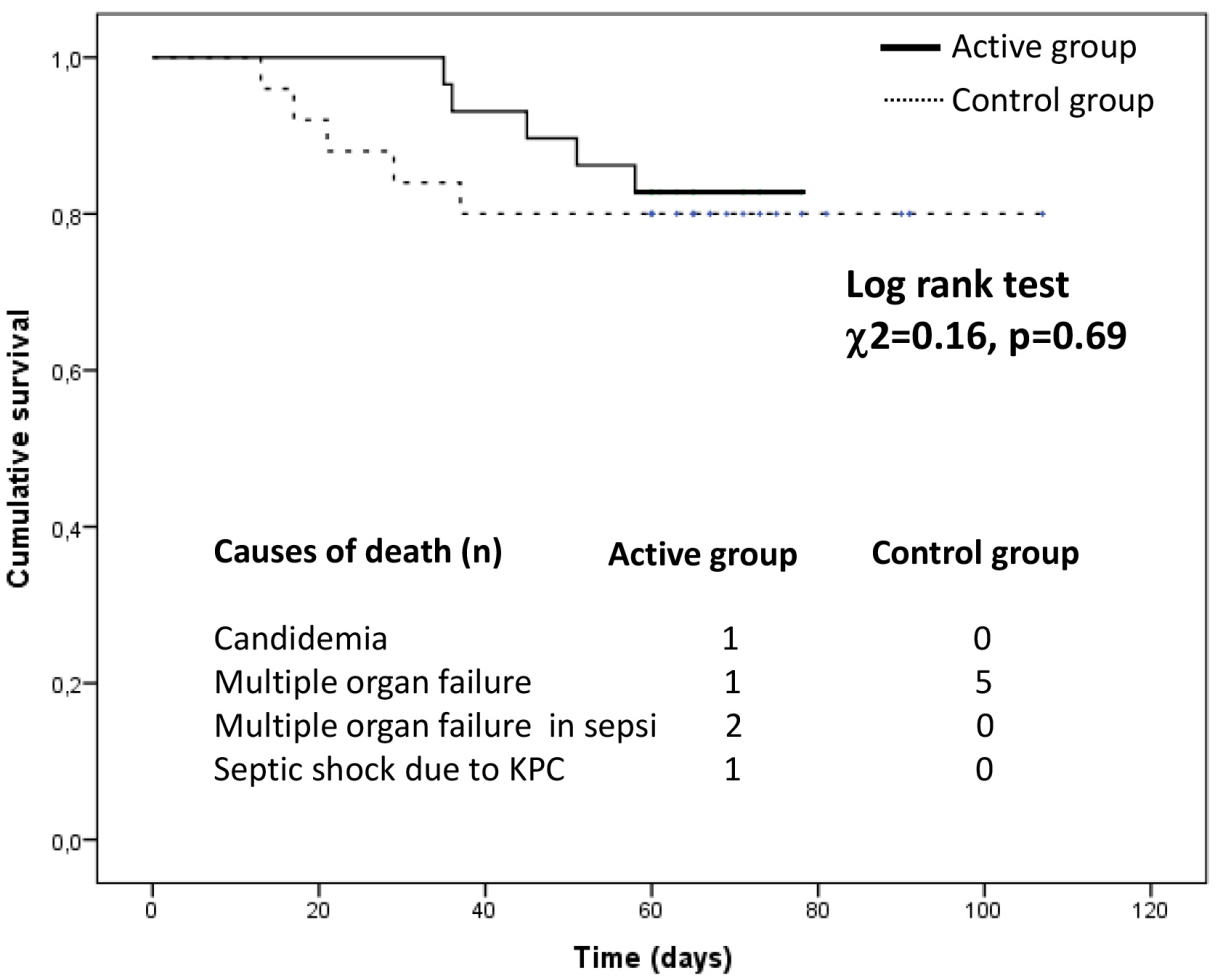
**Cumulative survival in the active group compared with the control group**.

During the follow-up period (median 60 days, inter-quartile range 60–71 days), 10 patients died. Among these, 5 deaths were observed in the active arm (17%) and 5 in the control arm (20%). In the active arm, causes of death were candidemia in 1 case, multiple organ failure in 1 case, multiple organ failure in sepsis in 2 cases, and septic shock due to Klebsiella pneumoniae in 1 case. In the control group, all death cases were due to multiple organ failure. In a Kaplan Meier survival analysis, the incidence of mortality did not significantly differ between patients in the active arm and those in the control arm (Log-rank test, χ^2^ = 0.16, *p* = 0.69) (Fig. 4).

## 4. Discussion

### 4.1 Main Findings

In this study, combining the standard treatment plus early adjuvant treatment with blood purification and IgM enriched immunoglobulins in postoperative cardiac surgical patients reduced biochemical and biohumoral variables over time. We also observed a restoration in the cardiovascular system balance, particularly considering the V/A coupling.

### 4.2 Sepsis in Cardiac Surgery

Although the low prevalence of sepsis in cardiac surgery (0.38–2%), the clinical consequences are higher mortality and significant prolonged ICU stay [45, 53, 54, 55, 56, 57] compared to sepsis in other patient populations. It is difficult to identify a specific cause that justifies the high mortality rate. Numerous studies have analyzed some factors: cardiovascular comorbidities typical of this population, the complexity of cardiac surgery procedures, and the use of cardiopulmonary bypass [58, 59, 60, 61, 62, 63, 64]. However, it is also known that gram-negative bacteria, frequently found in the gut flora, account for the majority of the early bloodstream infections of patients undergoing CPB, particularly those with extended durations [30, 65].

However, the most important factor correlated with mortality is the myocardial septic dysfunction in compromised patients. Sepsis-induced myocardial dysfunction is one of the significant predictors of morbidity and mortality of sepsis, and it is present in more than 40% of cases of sepsis. For this reason, in recent years, the attention of many researchers has shifted to mitigating the effect of mediators and cytokines on the cardiovascular system to treat and control septic myocardial dysfunction through newly developed techniques such as EBPT and intravenous immunoglobulins [66].

### 4.3 EBPT Combined with IgM-Enriched Immunoglobulin

Those new treatments today represent an essential tool for clinicians to minimize the peak of cytokine concentration, enhance the patient immune response and influence several stages of septic shock and multiorgan dysfunction. EBPT can affect both the trigger factors of sepsis by eliminating specific molecules (i.e., endotoxin) and, according to the peak concentration hypothesis’ of Ronco *et al*. [67], restore the immune balance by eliminating excessive pro- and anti-inflammatory cytokines. Notably, some authors report considerable decreases in the cytokine concentrations both after adsorption therapy and other after EBPT, including LPS adsorption that allows elimination of circulating proapoptotic factors and active mediators that would otherwise induce the injury of various organs and systems, as well as facilitate the recovery of the immune balance [68]. Pieces of evidence support the use of IgM-enriched immunoglobulins in a subgroup of patients with sepsis, showing improvement in survival [69, 70, 71], and when used prophylactically in patients undergoing procedures with cardiopulmonary bypass [72]. In patients with sepsis or septic shock, Domizi *et al*. [73] demonstrated that a 72-hour infusion of IgM-enriched immunoglobulins (Pentaglobin) may be associated with an increase in sublingual microvascular perfusion and that these changes did not correlate with variations in macro-hemodynamic parameters or cytokine levels. In our investigation, postoperative cardiac surgery patients who received combined extracorporeal therapy and IgM enriched immunoglobulins showed improvement in hemodynamics, although with no difference in vasopressor and inotropic support.

### 4.4 Endotoxin 

In literature, endotoxin has been shown to influence viscoelastic coagulation parameters, thus suggesting a link between endotoxin levels and the altered coagulation phenotype in septic patients [74].

Wand *et al*. [75] showed that the treatment with IgM-enriched immunoglobulin attenuates the EA levels in patients with severe sepsis and might reduce septic thrombocytopenia and fibrinogen depletion. However, viscoelastic, aggregometry or inflammatory parameters were not influenced.Unfortunately, the authors did not evaluate clinical outcomes. Although circulating endotoxins liberation is common during sepsis and its prognostic value is poor, there are also spontaneously elevated levels of IgM anti-endotoxin antibodies associated with a better outcome [76], and this effect can be replicated by the administration of IgM enriched immunoglobulins. However, the mechanisms of endotoxin neutralization and the efficacy of intravenous immunoglobulin treatment remain to be proved. The benefits of the EBPT and IgM enriched immunoglobulins in the present study to treat sepsis are demonstrated by the reduction of EAA, WBC, and SOFA scores. Our data were in line with a recent small study by Paternoster *et al*. [77] that reported the association with Ultraflux ® EMIC2 Filter and Pentaglobin to early treat septic shock after cardiac surgery to reduce the concentration of endotoxin activity safely. Although the small sample size, the incidence of mortality did not significantly differ between the two groups [77, 78].

### 4.5 Clinical Implications

Our results imply that targeted use of extracorporeal blood purification in combination with intravenous administration of IgM enriched Immunoglobulin (Pentaglobin®) 5 mL/kg die for at least three consecutive days and in conjunction with standard care approach in specific postoperative patient population improve the outcome in septic shock.

### 4.6 Strengths and Limitations

The present study is the first to provide data on extracorporeal blood purification in combination with intravenous administration of IgM enriched Immunoglobulin (Pentaglobin®) 5 mL/kg die for at least three consecutive days and in conjunction with a standard care approach, in a specific patients population. This study has several limitations. The retrospective and observational nature is the primary limit, and the small number of patients analyzed in the two groups. This last point, however, must be correlated with the low incidence of sepsis after cardiac surgery, which is now estimated at 5%. Therefore, our results should be considered exploratory and need confirmation by future studies. Our study was not powered to detect differences in mortality or other significant outcomes (organ failures, shock reversal, intensive care unit length of stay). Although the small sample size was a statistical challenge, it was adequately addressed using appropriate statistical methodology. Some variables (SOFA score, WBC, and the EAA) significantly differed between the active and the control group, but considering the non-randomized nature of the study, this is not a significant limitation. In addition, we did not measure baseline immunoglobulin levels, and we specified details of each EBPT used. Finally, our study did not contemplate a sample size calculation, a limitation that suggests caution when interpreting the study results, particularly those that did not achieve statistical significance.

## 5. Conclusions

The immunomodulation approach is a non-selective and broad-spectrum strategy to balance pro and antinflammatory mediators from the bloodstream and restore immune homeostasis. Few reports described successful combined treatment with blood purifications and IgM-enriched immunoglobulins in post-cardiac surgery septic shock. Blood purification and IgM enriched administration act as self-tailored therapies. Combined treatment reduced plasma levels of EAA, PCT, and IL-6 and improved cardiovascular performance by restoring V/A coupling and reducing inotropic score in the active group compared with the control group.

Even with some limitations, the present study suggests a potential beneficial effect of combined treatment with blood purification and IgM-enriched immunoglobulins on macrocirculation and cytokine modulation.

These preliminary data are promising results but need others to study and research. Although further well-designed randomized control trials are needed, this promising approach could represent new therapeutic options for septic patients after cardiac surgery.
